# Device-measured sedentary behavior and physical activity in older adults differ by demographic and health-related factors

**DOI:** 10.1186/s11556-020-00241-x

**Published:** 2020-06-11

**Authors:** Ing-Mari Dohrn, Paul A. Gardiner, Elisabeth Winkler, Anna-Karin Welmer

**Affiliations:** 1 Division of Physiotherapy, Department of Neurobiology, Care Sciences and Society, Karolinska Institutet, SE-141 83 Huddinge, Sweden; 2 Aging Research Center, Department of Neurobiology, Care Sciences and Society (NVS), Karolinska Institutet and Stockholm University, Stockholm, Sweden; 3 Centre for Health Services Research, The University of Queensland, Brisbane, Australia; 4 School of Public Health, The University of Queensland, Brisbane, Australia; 5 Allied Health Professionals, Function Area Occupational Therapy & Physiotherapy, Karolinska University Hospital, Stockholm, Sweden; 6 Stockholm Gerontology Research Center, Stockholm, Sweden

**Keywords:** Accelerometery, Cohort study, Sitting, Validation

## Abstract

**Background:**

Our aim was to describe and explore older adults’ device-measured sedentary behavior and physical activity (PA) pattern by sex, age, education, marital status, body mass index, and physical function; and to assess agreement regarding fulfillment of PA recommendations, i.e. 150 min/week of moderate-to-vigorous intensity PA (MVPA), between device-measured and self-reported PA.

**Method:**

We included 656 older adults (64% women), aged 66, 81–87 or ≥ 90 years from a Swedish population-based cohort study. The activPAL3 accelerometer provided information on sedentary behavior (sedentary time, sedentary bouts, sit-to-stand transitions) and PA. Stepping ≥100 steps/min was considered MVPA; standing and stepping < 100 steps/min were considered light-intensity PA (LPA). Self-reported PA was compared with min/week in MVPA and steps/day.

**Results:**

On average, 60% of wear time was spent sedentary, 36% in LPA, and 4% in MVPA. Relative to men, women, had significantly (*p* < 0.05) more sit-to-stand transitions, spent 33 min/day less sedentary and 27 min/day more in LPA, and were more likely to report meeting PA recommendations, but showed no difference in steps/day, MVPA, or sedentary bout duration. Older age was associated with more sedentary time, lower MVPA and fewer steps/day. The prevalence of meeting PA recommendations was 59% device-measured and 88% by self-report with limited agreement between methods (Cohen’s Kappa = 0.21, Spearman’s rho = 0.28). Age differences were much more pronounced with objective measures than by self-report.

**Conclusions:**

We found significant sex differences in sedentary behavior and time in LPA in older adults, but not in MVPA, in contrast to previous findings. Sedentary time increased with age, with small differences in accumulation pattern. MVPA time was lower with older age, obesity, and poor physical function. A majority of the participants > 80 years did not meet the PA recommendations. Given the strong relationships between sedentary behavior, PA and health in older adults, programs are needed to address these behaviors. Agreement between device-measured and self-reported fulfillment of PA recommendations was limited. Device-based measurement adds value to PA studies, providing richer and different data than self-report.

## Introduction

Physical activity (PA) is essential for healthy aging. Older adults who are physically active have lower rates of all-cause mortality and many chronic diseases, a lower risk of falling, better physical function and better cognitive function [1]. The robust evidence on the health benefits of PA has led to public health PA guidelines recommending that adults ≥65 years should accumulate at least 150 min/week of moderate-to-vigorous intensity PA (MVPA), i.e. ≥3 metabolic equivalents (METs) [1], with brisk walking often used as a primary example of an appropriate activity. In addition, muscle strengthening activities should be performed two or more days per week. Yet, adherence to PA recommendations is low in older adults, with studies relying on self-reported data indicating that 55 to 70% do not achieve the minimum level of MVPA [2, 3].

Additionally, older people may spend up to 80% of their waking day sedentary [4–9] and the sedentary time seems to increase with age. Growing scientific evidence has recognized that sedentary behavior is a distinct risk factor for poor health, including increased risk of mortality, type 2 diabetes, metabolic syndrome, cardiovascular disease and cancer [10–12]. Sedentary behavior is measured in varied ways but consistently defined as any waking behavior characterized by an energy expenditure ≤1.5METs, while in a sitting, reclining or lying posture [13]. Research suggests that not only the total time spent sedentary is of importance, but also the pattern of how the sedentary time is accumulated, i.e., prolonged vs shorter sedentary bouts or how frequently sedentary time is interrupted. Interruptions to sedentary time (breaks) play an important role for cardio-metabolic health [14, 15], and may also be associated with muscle mass and physical function in older adults [16–18].

It is known that self-reported data have low accuracy due to recall bias or social desirability. Sedentary time is often underestimated and MVPA is often overestimated, while light-intensity PA (LPA), which is common among older adults, is almost impossible to recall or report accurately [19–21]. It is therefore highly relevant to use device-based methods, which more accurately can capture the whole PA intensity spectrum, as well as other aspects of the daily movement pattern, for example sedentary time in bouts. Body-worn activity monitors, such as accelerometers, are shown to be sensitive and feasible for measuring PA and sedentary behavior in older adults, although it has been sparsely used in the oldest age groups. Studies that have used accelerometry to assess PA and sedentary behavior in individuals over 70 years have found that patterns differ according to gender, age group, education, and body mass index (BMI) [4, 6–9, 22].

To date, most evidence on associations between device-based sedentary behavior and health has derived from accelerometers defining sedentary behavior as lack of movement, in contrast to measures derived from actual sitting posture. This can lead to misclassification of low-intensity non-sedentary behavior, such as standing [23]. The thigh-mounted activPAL3 is an accelerometer that directly measures the postural aspect of sedentary behavior, i.e. sitting/lying vs upright position, and thereby also more accurately can capture breaks in sedentary time by registering sit-to-stand transitions [23]. Richer knowledge of older adults’ pattern of sedentary time accumulation and PA in both light and moderate-to-vigorous intensity can guide development of health promoting efforts tailored for older people. Therefore, our aims were to describe and explore older adults’ device-measured sedentary behavior and PA pattern by sex, age, education, marital status, BMI and physical function; and to compare agreement regarding fulfillment of PA recommendations between device-measured and self-reported PA.

## Method

### Study population and data collection

We used data from the ongoing population-based Swedish National study on Aging and Care in Kungsholmen (SNAC-K) study. The study sample consists of persons randomly selected from eleven age groups ≥60 years living at home or in nursing homes in Kungsholmen, Stockholm. Details of the original sampling methods and response rates are presented elsewhere [24]. Briefly, n = 3363 (73% of eligible people) in the ages 60, 66, 72, 78, 81, 84, 87, 90, 93, 96, and 99 participated in the baseline examination 2001–2004. Follow-up examinations are performed every six years for younger cohorts (60–78 years) and every three years for older cohorts (≥78 years). At the follow-up examinations 2016–2018, n = 1287 participants in the age groups 66, 81, 84, 87, 90 and ≥ 93 years were examined. Out of those, n = 680 participants who were eligible (no severe cognitive impairment; able to move indoors without assistance) agreed to wear an accelerometer the following week and comprise the study population in this study. Data were collected through interviews and tests at the SNAC-K research center or in the participants’ homes by trained staff.

### Socio-demographic and health variables

Age, sex, education (less than high school, high school, or university) and marital status (i.e. married/living together (yes/no), and use of walking aid (yes/no) were derived from interviews. BMI was calculated from weight and height measured using standard methods and categorized as underweight < 20, normal 20–24, overweight 25–29, and obese ≥30 kg/m^2^, adapted for older adults [25]. For the analyses of age-related differences in sedentary behavior and PA we categorized participants into three age groups: 66 years, 81–87 years, and ≥ 90 years.

### Physical function

Physical function was assessed with the 5 times sit to stand test (5 STS). The 5 STS was performed by asking the participants to stand up and sit down five times as fast as they could, without using the arms, and categorized as ability to perform five consecutive chair stands (yes/no) [26]. In addition, the balance test one-leg stance (OLS), eyes open, was used to describe the sample. Each leg was tested twice, and the best overall score was used and categorized as ability to stand ≥5 s (yes/no) [27].

### Self-reported PA

Self-reported PA derived from two questions in a self-administered questionnaire distributed by the study nurse to be completed by the participants after the examination [28]: 1) “Do you regularly engage in light exercise? (Walking on roads or in parks, walking in the woods, short bicycle rides, light aerobics, golf)” and 2) “Do you regularly engage in more intense exercise? (Jogging, brisk long walks, heavy-duty gardening, long bicycle rides, high-intensity aerobics, long distance ice skating, skiing, swimming, ball sports (not golf) or other similar activity)”. For both questions the answer alternatives were; “In the last 12 months: every day, several times/week, 2-3 times/month, less, never.” The answers from the two PA questions were collapsed into a dichotomous variable (yes/no) with reference to the recommended PA for health [1]. Participants were considered meeting recommendations if they were engaged in light and/or intense exercise every day or several times per week, and not meeting recommendations if they chose the other response options.

### Assessment of sedentary behavior and PA

The activPAL3 accelerometer (PAL Technologies Ltd., Glasgow, UK) was used to assess sedentary behavior and PA. The activPAL3 is a small and slim thigh-worn activity monitor that uses triaxial acceleration to determine thigh angle and thus body posture (sitting/lying or upright), along with transitions between these postures, and stepping speed (cadence), from which energy expenditure can be estimated. The activPAL3 has high accuracy for measuring time spent sedentary (i.e., sitting/lying), standing or stepping, and for pattern of sedentary time accumulation [23]. Participants were asked to continue with their usual PA habits while wearing the activPAL3 for seven consecutive days during all waking hours (excluding showering and swimming), starting the day after the examination, and to record time when they put on and removed the device each day on a log-sheet. The study nurse provided verbal, visual, and written instructions to participants during the examination at the research center or home visit on how to wear the device correctly. The participants were provided with a Micropore 2.5 cm tape for sensitive skin to adhere the activPAL3 to the thigh and with a prepaid envelope to return the device by mail.

### Data processing activPAL3

The activPAL3 files were processed using the PALbatch software, using a feature to correct for upside down wear. A custom-made syntax for SAS programming system was used for further analyses of the events files from the PALbatch software and to remove log-reported non-wear time [29]. Consistent with recommendations for the field [23], visual examination of heat maps was performed to check consistency between recorded movement and the times provided in the log-sheet, making corrections when appropriate (e.g., participant had confused AM and PM) and estimating missing non-wear or sleep times. Accelerometer data of participants’ with least four valid measurement days, were included in the data analysis. A measurement day was considered valid if wear time was at least 10 h during waking hours.

Prior work has shown that 100 steps/min is an appropriate threshold value for 3 METs [30], consequently, we used cadence of ≥100 steps/min for MVPA, and total time spent either stepping at a cadence < 100 steps/min or standing for LPA. Variables from the activPAL3 were: number of steps/day, daily time in MVPA and daily time in LPA (defined as described above), daily sedentary time, number of sit-stand transitions per sedentary hour (a similar concept to breaks in sedentary time), usual bout duration (midpoint of the cumulative distribution of sedentary bout durations, i.e. half of all sedentary time is accumulated in bouts longer than the usual bout duration) [31], and longest sedentary bout.

For tests of agreement with self-reported PA, three dichotomous variables (yes/no) were created from the activPAL3 data to classify if a person was considered to meet the aerobic PA recommendations. The first variable derived from the median daily time spent in MVPA multiplied by seven and participants with at least 150 min/week of stepping with a cadence ≥100 steps/min were considered to have met PA recommendation. Neither prolonged nor regular activity during the week were required. The other two variables derived from the daily step count with cutoffs set at 7000 and 5000 steps/day. A level of approximately 7000 steps/day has been suggested to equal ≥150 min/week MVPA in older adults [32, 33]. However, 7000 steps/day may be a too high goal for some older adults, especially in the oldest age groups. Therefore, we also compared with 5000 steps/day, which may be a more realistic level and a level shown to relate to better health outcomes [34].

### Statistical analyses

Descriptive data are presented as numbers and proportions (%), mean and standard deviation (SD), or median and interquartile range. Due to skewed MVPA data, we used multivariate quantile regression for continuous variables to assess differences in daily movement pattern within subgroups reported as adjusted average median and 95% confidence intervals (CI) of individual means. For dichotomous data we used multivariate logistic regression presented as adjusted proportions of individual means or odds ratios and CI. Covariates included in the models were sex, age group, education, marital status, BMI, physical function, and accelerometer wear time.

The relationships between self-reported and accelerometer derived data were calculated with Cohen’s kappa to determine the kappa coefficient (K), with confidence intervals (CI) and Spearman’s correlations (rho). The kappa coefficient adjusts for the agreement attributable to chance and is recommended when validating PA questions with categorical measures [35]. Statistical significance was defined as two-tailed *p* < 0.05. Analyses were computed in Stata 15.1 (StataCorp LP, College Station, TX, USA) and SAS 9.4 (SAS Institute Inc., NC, USA).

## Results

### Participant characteristics

Out of the 680 persons who wore an accelerometer, 24 persons were excluded due to either device malfunction (n = 4), device not worn according to the instructions (n = 2) or < 4 valid days (n = 18), leaving an analytical sample of n = 656. Compared to the participants that were excluded or declined to wear the device, the analytical sample included a larger proportion of the 66 years age group (58% vs 26%) and a smaller proportion of the 81–87 and ≥ 90 years age groups (37% vs 50 and 4.5% vs 24%). There we no differences in the proportion of women between the excluded and analytical sample (both 64%). The majority of the participants (78%) had seven accelerometer wear days and mean wear time per day was 14 h and 12 min. Due to missing self-reported data, the sample size for analyses of agreement between device-measured and self-reported PA was n = 619.

### Sedentary behavior and PA by subgroups

Table 1 shows participant characteristics and crude daily movement pattern presented as percentages of wear time spent sedentary, in LPA, and in MVPA, and mean steps/day for all study participants and stratified by sex and age group. Median daily time sedentary was 8 h 32 min, 5 h 2 min in LPA, and 26 min in MVPA for the total sample. Steps/day ranged from 987 to 23,475 (min-max), with a median of 8371. A majority of the day was spent sedentary in both women and men, and across age groups, with group means ranging from 58 to 67% of wear time. Proportion of time in LPA ranged from 31% in the ≥90 group to 37.5% among those with normal BMI, with small but significant differences in all subgroups except education. Proportion of time in MVPA ranged from 1.6–4.6%, with least activity registered in the ≥90 group and the highest in 66 year olds. Women spent median 33 min less sedentary and 27 min more in LPA than men, while MVPA or steps/day did not vary statistically significant by sex.

**Table 1:** Characteristics of the total study population and by sex and age group

	Total (n = 656)	Women (n = 420)	Men (n = 236)	66 y (n = 383)	81–87 y (n = 243)	≥90 y (n = 30)
**Age**	73.3 (±9.0)	74.1 (±9.3)	71.7 (±8.2)	66.0 (±1.0)	82.6 (±2.3)	91.1 (±2.3)
**Sex**						
Women	420 (64)	n/a	n/a	228 (59)	165 (68)	17 (90)
**Education**						
University	375 (57)	223 (53)	152 (64)	255 (66)	111 (46)	9 (30)
**Marital status**						
Married/living together	367 (56)	193 (46)	174 (74)	252 (66)	113 (46)	2 (7)
**Body mass index**						
Underweight, < 20	25 (4)	24 (6)	1 (0.5)	9 (2)	14 (6)	2 (7)
Normal, 20–24	287 (44)	200 (48)	87 (37)	171 (45)	102 (42)	14 (47)
Overweight, 25–29	254 (39)	140 (33)	114 (48)	151 (39)	92 (38)	11 (37)
Obese, ≥30	89 (13)	55 (13)	34 (14)	52 (14)	34 (14)	3 (10)
**Physical function**						
One-leg-stance ≥5 s	587 (90)	292 (72)	180 (79)	354 (92)	110 (49)	8 (28)
5 STS test, able to	472 (74)	372 (89)	215 (92)	375 (98)	197 (82)	15 (52)
Use walking aid	82 (10)	69 (17)	13 (5.5)	7 (2)	57 (24)	18 (60)
**Physical activity**						
Sedentary, % of wear time	60.2 (±10.8)	59.1 (±10.4)	62.1 (±10.8)	58.3 (±10.9)	62.2 (±9.9)	67.3 (±11.1)
Light PA, % of wear time	36.1 (±10.1)	37.3 (±9.9)	34.1 (±10.1)	37.0 (±10.4)	35.4 (±9.2)	31.1 (±10.6)
MVPA, % of wear time	3.6 (±3.0)	3.6 (±2.9)	3.7 (±3.1)	4.6 (±3.0)	2.4 (±2.3)	1.6 (±2.8)
Steps/day	8696 (±3779)	8571 (±3820)	8918 (±3704)	10,106 (±3573)	6952 (±3011)	4818 (±3390)
Accelerometer wear time, min/day	852 (± 64)	850 (± 62)	857 (± 66)	866 (± 62)	834 (± 60)	831 (± 66)
Self-reported regular^a^ MVPA^b^	542 (88)	353 (88)	189 (86)	326 (90)	194 (85)	22 (76)

Data are reported as numbers (%) or mean (±SD). ^a^Every day or several times/week, ^b^n = 619. MVPA = moderate-to-vigorous physical activity; PA = physical activity; 5 STS = 5 times sit to stand test

In Table 2, sedentary behavior among all study participants and stratified by subgroups is presented. Women had more sit-to-stand transitions per hour of sedentary time than men, but there were no significant sex differences regarding sedentary bout duration. Older age was associated with more sedentary time, fewer sit-to-stand transition and longer usual sedentary bout. The length of usual sedentary bout ranged from 8 to 84 min, and only 5% of the sample had a usual sedentary bout over 80 min. Individuals who were unable to do 5 STS spent 40 min/day more sedentary and had fewer sit-to-stand transitions compared with those who were able to do 5 STS, on average.

**Table 2:** Sedentary behavior among all study participants and in subgroups

	Sedentary time (min/day)	Sit-to-stand transitions (n)	Usual sedentary bout (min)	Longest sedentary bout (min)
Total sample^a^	512.1 (455.6–571.7)	5.1 (4.0–6.4)	30.1 (24.4–39.1)	132.6 (106.4–167.2)
**Sex**				
Women	502.6 (492.2–513.1)*	5.3 (5.1–5.5)*	30.5 (28.8–32.1)	135.0 (128.1–141.9)
Men	535.6 (521.6–549.9)	4.8 (4.5–5.1)	31.3 (30.1–32.4)	133.1 (128.0–138.2)
**Age group**				
66 years	504.5 (493.3–515.8)*	5.4 (5.2–5.6)*	29.9 (28.6–31.2)*	133.2 (127.7–138.7)
81–87 years	526.0 (511.9–540.1)	4.8 (4.5–5.0)	32.0 (30.3–33.6)	134.3 (127.4–141.2)
≥ 90 years	552.3 (510.6–593.9)	4.5 (3.7–5.3)	37.4 (32.6–42.3)	137.4 (117.0–157.8)
**Education**				
≤ High school	516.5 (503.8–529.3)	5.0 (4.8–5.3)	31.4 (30.0–32.9)	133.7 (127.4–139.9)
University	513.0 (502.1–524.0)	5.2 (5.0–5.4)	30.7 (29.4–32.0)	133.9 (128.5–139.2)
**Marital status**				
Living alone	526.2 (513.3–539.0)*	5.0 (4.8–5.3)	31.4 (29.8–32.9)	134.7 (128.4–141.0)
Married/partner	505.4 (494.1–516.7)	5.2 (4.9–5.4)	30.7 (29.4–32.0)	133.1 (127.6–138.6)
**Body mass index**				
Underweight < 20	512.0 (469.7–554.4)	5.6 (4.8–6.4)*	26.9 (22.0–31.9)*	130.3 (109.6–151.0)
Normal 20–24	501.5 (489.0–513.9)	5.4 (5.2–5.7)	29.0 (27.6–30.5)	130.0 (124.0–136.1)
Overweight 25–29	520.6 (507.4–534.0)	5.0 (4.7–5.2)	32.0 (30.5–33.6)	134.8 (128.3–141.3)
Obese ≥30	539.6 (517.4–561.8)	4.4 (4.0–4.8)	35.5 (32.9–38.1)	143.7 (132.9–154.6)
**Physical function**				
5 STS, not able to	550.1 (521.9–578.2)*	4.3 (3.8–4.9)	35.3 (32.0–38.6)*	144.4 (130.6–158.1)
5 STS, able to	510.6 (502.0–519.3)	5.2 (5.0–5.4)	30.5 (29.5–31.5)	132.6 (128.4–136.9)

Data are reported as average median (confidence intervals), adjusted for all other listed variables plus wear time, *p < 0.05 within subgroups. ^a^Unadjusted median and interquartile range. 5 STS = 5 times sit to stand test

Time spent in MVPA and fulfillment of MVPA recommendations are shown in Table 3 for all study participants and by subgroups. The prevalence of meeting PA recommendations ranged from 59% (≥150 min/week stepping at ≥100 steps/min cadence) to 88% (self-reports being active in MVPA every day or several days/week). Median MVPA time was significantly lower to a large degree with older age, obesity, and poor physical function, and significantly lower to a smaller degree with less education. These patterns were largely reflected in the patterning of prevalence of meeting physical activity recommendations, though not all differences were statistically significant. Age differences were much more pronounced with the objective measures than by self-report. Additionally, relative to men, women were significantly more likely to meet ≥150 min/week MVPA recommendation (64% vs 54%) with smaller and non-significant differences by sex seen for the other measures. Differences by marital status were small (< 10 min, < 10%), and were significant only for meeting ≥5000 steps/day.

**Table 3:** Time in MVPA and fulfillment of different defined MVPA recommendations

	MVPA min/d	≥ 150 min/w MVPA	≥ 7000 steps/d	≥ 5000 steps/d	Self-reported regular PA^b^
Total sample^a^	25.8 (10.9–44.8)	59	64	84	88
**Sex**					
Women	27.3 (24.9–29.8)	64*	67	91	91
Men	23.9 (20.6–27.2)	54	66	89	86
**Age group**					
66 years	32.4 (29.7–35.1)*	75*	78*	95*	90
81–87 years	17.6 (14.3–20.9)	38	51	79	88
≥ 90 years	12.5 (2.7–22.3)	23	22	61	86
**Education**					
≤ High school	23.7 (20.7–26.7)*	55*	56*	88	86*
University	27.8 (25.3–30.4)	64	74	91	91
**Marital status**					
Living alone	25.4 (22.3–28.4)	62	63	87*	86
Married/partner	26.6 (24.0–29.3)	59	70	92	91
**Body mass index**					
Underweight < 20	36.7 (26.8–46.7)*	85*	70*	95*	89
Normal 20–24	29.4 (26.5–32.3)	69	76	93	91
Overweight 25–29	24.8 (21.7–27.9)	58	65	88	89
Obese ≥30	16.1 (10.9–21.4)	29	38	80	77
**Physical function**					
5 STS, not able to	15.7 (9.1–22.3)*	33*	49	78*	79*
5 STS, able to	27.2 (25.2–29.3)	63	67	91	90

Data are reported as average median (confidence interval) or proportions in %, adjusted for all other listed variables plus wear time, *p < 0.05 within subgroups. ^a^Unadjusted median (interquartile range). ^b^n = 619. MVPA = moderate-to-vigorous physical activity; 5 STS = 5 times sit to stand test

### Agreement regarding fulfillment of PA recommendations

Table 4 shows the concurrent validity for self-reported PA vs accelerometer derived PA for identifying participants classified as sufficiently active according to PA recommendations, expressed as Spearman’s rho (relative validity) and Cohen’s kappa coefficient (agreement). In the total sample, agreement between self-report and device-measured ≥150 min/week MVPA was fair (Κ = 0.21) [36], and the correlation was weak (rho = 0.28) but significant. Similar results were seen with the other criteria. The overall validity was not reflective of the results seen in each subgroup, with especially low validity seen in the oldest age group and those unable to perform the 5 STS test (particular in terms of kappa, which is chance-corrected), and with especially good validity seen in underweight participants (BMI < 20). Both relative validity and agreement were higher in men than in women. The kappa agreement for ≥150 min/week MVPA was highest in the 66 years age group and in participants living alone (K = 0.25). Agreement between self-report and steps/day was higher than for ≥150 min/week MVPA overall.

**Table 4:** Concurrent validity for self-reported physical activity and accelerometer derived physical activity

Characteristics (n)	≥ 150 min/week MVPA stepping^a^		≥ 7000 steps/day		≥ 5000 steps/day	
	Spearman’s rho	Kappa (95% CI)	Spearman’s rho	Kappa (95% CI)	Spearman’s rho	Kappa (95% CI)
All (619)	0.28	0.21 (0.15–0.28)	0.31	0.25 (0.18–0.32)	0.32	0.32 (0.22–0.42)
Women (400)	0.27	0.20 (0.13–0.28)	0.30	0.23 (0.15–0.31)	0.31	0.30 (0.18–0.42)
Men (219)	0.29	0.22 (0.12–0.32)	0.34	0.30 (0.17–0.42)	0.36	0.36 (0.19–0.54)
**Age group**						
66 years (361)	0.29	0.25 (0.14–0.36)	0.35	0.32 (0.20–0.44)	0.31	0.29 (0.12–0.46)
81–87 years (229)	0.24	0.14 (0.07–0.21)	0.25	0.17 (0.08–0.25)	0.32	0.30 (0.16–0.44)
≥ 90 years (29)	0.26^#^	0.12 (−0.01–0.25)	0.22^#^	0.10 (−0.01–0.21)	0.27^#^	0.20 (−0.04–0.45)
**Education**						
≤ High school (268)	0.28	0.22 (0.13–0.30)	0.25	0.19 (0.10–0.27)	0.38	0.37 (0.23–0.51)
University (351)	0.25	0.19 (0.10–0.27)	0.35	0.29 (0.18–0.40)	0.23	0.23 (0.08–0.38)
**Marital status**						
Living alone (275)	0.32	0.25 (0.15–0.34)	0.30	0.23 (0.14–0.32)	0.31	0.30 (0.17–0.44)
Married/partner (344)	0.23	0.17 (0.08–0.25)	0.31	0.25 (0.15–0.35)	0.31	0.32 (0.16–0.47)
**Body mass index**						
Underweight < 20 (22)	0.42^#^	0.40 (−0.07–0.87)	0.53	0.43 (0.07–0.79)	0.23^#^	0.23 (−0.31–0.76)
Normal 20–24 (278)	0.25	0.19 (0.09–0.30)	0.24	0.20 (0.08–0.31)	0.39	0.38 (0.21–0.56)
Overweight 25–29 (233)	0.23	0.16 (0.07–0.26)	0.30	0.23 (0.12–0.34)	0.29	0.28 (0.12–0.44)
Obese ≥30 (85)	0.32	0.21 (0.09–0.33)	0.33	0.25 (0.10–0.39)	0.24	0.24 (0.01–0.47)
**Physical function**						
5 STS, not able to (59)	0.23^#^	0.14 (0.01–0.26)	0.20^#^	0.13 (−0.02–0.28)	0.39	0.35 (0.14–0.57)
5 STS, able to (555)	0.27	0.20 (0.13–0.27)	0.31	0.25 (0.17–0.32)	0.28	0.28 (0.16–0.39)

^a^MVPA stepping: ≥100 steps/min. ^#^Non-significant. CI = confidence interval; MVPA = moderate-to-vigorous physical activity; 5 STS = 5 times sit to stand test

## Discussion

In this study aiming to describe and explore older adults’ device-measured pattern of sedentary time and PA by subgroups, we found that women spent about 30 min less sedentary and equivalent more time activities of light intensity, such as household chores or slow walking. These sex differences are in line with similar populations in Europe [4, 8, 9] and may be of importance for health. Previous research has shown that substituting 30 min of sedentary time to LPA may reduce the risk of all-cause mortality with 11% and risk of cardiovascular disease with 24% [37]. Interestingly, there were only small sex differences in pattern of sedentary time accumulation. Total sedentary time was higher with increasing age. Pattern of sedentary time accumulation varied by age group, but the differences in number of sit-to-stand transitions and usual sedentary bout duration were quite small, with no differences in longest sedentary bout. Sedentary behavior did not vary by education or marital status except for total sedentary time, which was 20 min/day more in those who were living alone. Obese participants spent 40 min/day more sedentary than those with normal weight on average and they also had a less favorable sedentary accumulation pattern. These differences were also seen for individuals with impaired physical function.

We did not find statistically significant sex differences in time spent in MVPA or number of daily steps. This is in contrast to many other studies, both those using self-reports and accelerometers, which found males to be significantly more active than females [38]. It is known that many PA questionnaires give examples of MVPA activities that traditionally are mostly performed by men, especially in older generations, and therefore are likely to underestimate MVPA in older women. However, recent device-based studies have also found that MVPA is higher in men [4, 8, 39, 40]. In our study, on the contrary, we found that women were more likely to fulfil the PA recommendations of 150 min/week of MVPA than men in the adjusted models. Similar findings have previously been reported in two Norwegian studies [9, 41], and the discrepancies might depend on cultural and sociodemographic differences between older adults in the Nordic countries and other study populations.

As reported in previous research [4, 8], overweight and obese participants spent significantly less time in MVPA and had lower fulfillment of MVPA recommendations, compared to normal weight participants. Surprisingly, our underweight participants had a high level of MVPA. Underweight in older adults is often related to frailty and a lower muscle mass, which would be expected to result in lower MVPA. However, it is likely that our finding is a consequence of selection bias, and that our small sample of underweight participants, 24 woman and 1 man, consists of well-functioning, active older adults. Education level is another known correlate of PA [42], and this is consistent with what we found for MVPA, but not for sedentary behavior or LPA.

In our study sample, 26% of the participants were not able to do five chair raises without help from their arms. These older adults were significantly less active than those who could perform the test, with over 9 h/day sedentary and only 15 min/day in MVPA, which is clearly under the recommended PA level. Importantly, we also found that they had a lower number of sit-to-stand transitions and longer usual sedentary bout, suggesting that difficulty in raising from a chair may lead to a more sedentary behavior. Even though studies have found that breaks in sedentary time are associated with better health outcomes [16, 18], evidence is still inconclusive and more research, including prospective studies, is needed to gain a deeper understanding of these associations. Considering the negative health effects of prolonged sedentary behavior and a low PA level, it should be highlighted that this is a group of older adults that may benefit from interventions aiming to decrease sedentary time and increase MVPA. For example, a replacement of just 10 min of sedentary time with MVPA could result in a 34% reduction of cardiovascular mortality risk [37].

In agreement with other studies [8, 43], we found a wide variation in time in MVPA and steps/day within all age groups, indicating that high levels of PA may be sustained even among the oldest old. Two participants in the ≥90 group had a daily average of > 10,000 steps. However, when considering the data from study participants in this oldest age group, it is important to acknowledge that the results reflect those who have successfully aged and are able and willing to participate, and thereby represent a selective sample. Still, there is limited data on PA and sedentary behavior in the oldest old, and as the number of nonagenarians and centenarians are increasing worldwide, our results may contribute with valuable information about this group.

Walking is the most popular and most commonly reported form of physical activity in older adults, and to provide generalized guidance of the desired daily activity to meet the recommendations of 150 min/week of MVPA, a level of approximately 7000 steps/day has been suggested by the American college of Sports Medicine [33]. However, recognizing that the normative range for number of steps/day for older adults is 2000–9000 [32] and that many older adults may be limited in their everyday activities due to mobility limitations and chronic illness, we chose also to compare with 5000 steps/day as the threshold.

The age-related prevalence of meeting the PA recommendations of 150 min/week of MVPA ranged from 75% in the 66 years group to 23% in the ≥90 group, with very similar results if the 7000 steps/day definition was used. The few previous studies that have assessed device-measured PA in older adults divided by age groups have also shown that PA decreased progressively with age for both men and women [9, 38]. If the 5000 steps/day level was used, 95% in the 66 years group met the goal and 61% in the ≥90 group. This suggests that many of the oldest old, may find the recommendations challenging, and highlights the importance of a broad perspective when counselling older sedentary individuals [44]. A focus on the message to reduce sedentary time and increase light activities may be more realistic and pave the way to physical activity with higher intensity. The dose-response relation between physical activity and health clearly shows that there are benefits from any level of PA, and more is better [10].

The kappa agreement between self-reported and device-based MVPA was fair overall [36], which is in line with other studies. Previous validations of one or two PA questions using accelerometry assessed MVPA have found correlations of rho = 0.31 [45] and rho = 0.38 [46], and K = 0.18 [46]. Even more comprehensive PA questionnaires, and thereby possibly more accurate, have limited correlations in older populations ranging from rho = 0.11–0.65 [19].

Validation studies of self-report measures of sedentary time (usually ‘sitting’) have shown it is difficult for older people to accurately recall their behavior [19, 20]. Wearing devices can give people an accurate estimate of their behavior and therefore shape how they choose to respond to messages to sit less (as in the physical activity guidelines of many countries) and to ascertain the effectiveness of any changes to reduce their sedentary behavior. The same is true for PA. If we can develop better methods of self-report or cheaper devices that accurately measure these behaviors, then individuals can choose how to respond to that information. Our results add to the current knowledge that older adults sit for long periods and have low levels of PA, which are both suitable targets for change.

This study has several strengths. One important strength is the use of the activPAL3 accelerometer, which is a posture-based activity monitor. By using this device, we could investigate pattern of sedentary time accumulation in a more reliable way than studies using hip or wrist worn accelerometers, which estimate sedentary time based on limited acceleration rather than posture. The activPAL3 has also shown good accuracy in measuring steps in older adults who walk slowly and use an assistive device [47]. It might be worth noticing that the sedentary time derived from the activPAL3 can include reclining or lying during waking hours; however, these positions are included in the definition of sedentary time [13]. Another important strength is the population-based study sample, including 133 participants over 84 years. Further, a sample size of at least 50 participants has been suggested for validation studies on PA questionnaires [48]. The sample size in our study was over 650 participants, which made it possible to draw stronger conclusions, and to perform stratified analyses for subgroups. We used Cohen’s kappa for reporting results on the validity of the PA questions which is a considered a better choice of method for categorical measures than the often-used Spearman’s rho [19, 35].

A limitation of our study is that the design did not allow us to include participants with cognitive impairment or mobility limitations, leading to a study sample healthier than the general older population. It is also a limitation that the cadence of ≥100 steps/min used to define MVPA has not been validated for older adults, and some misclassification may have occurred. Ideally PA intensity cut points should be individually calibrated; nevertheless, this is not feasible in large study samples, a limitation shared with similar research. As in many other accelerometer studies, activities such as cycling, strength training, water-based activities, upper body movements and the effort of carrying loads were not captured. Additionally, even though we made a large effort to estimate wear time correctly, it must be acknowledged that the longest sedentary bout variable is sensitive to miscalculation of wear time. The overall wear time was high but is still likely to have missed some of all activities, since a 24-h wear protocol was not used, and participants did not always attach the devices immediately upon rising or immediately before bed. Further, our self-reported data provided information regarding the intensity and frequency of PA, but not the duration of the activity. Still, it is hard to accurately recall how many minutes that are spent in different activities in a day or week, especially unstructured activity interspersed over the day. Regular activities at a moderate intensity with longer durations are easier to recall and it is reasonable to assume that people reporting being active every day or several times per week also achieve the recommended PA level. The device-measured PA was collected over the entire year for different individuals and would thereby not be affected by season on a group level. Still, it is a limitation to the study that device-based PA was assessed over one week, while self-reported PA was asked about for the past 12 months. It should also be noted that, even though it is included in the PA recommendations [1], we did not investigate muscle strengthening physical activity. Finally, due to the cross-sectional design of this study we cannot draw any causal conclusions from the results.

## Conclusions

We found significant sex differences in daily sedentary behavior and LPA in older adults, but not in MVPA, in contrast to previous findings in self-reports. Women spent on average about 30 min/day less sedentary and equivalent more time performing activities of light intensity than men. Sedentary time increased with older age, with small differences in pattern of sedentary time accumulation. Individuals with limitations in physical function spent over 9 h/day sedentary and only 15 min/day in MVPA. A majority of the participants over 80 years did not meet the PA recommendations of 150 min/week of MVPA. Age differences were much more pronounced with objective measures than by self-report. The limited agreement between device-measured and self-reported fulfillment of PA recommendations illustrate the consequences of using different methodological approaches in PA surveillance. Device-based measurement adds value to studies, providing richer and different data than self-report, such as more detailed amounts and sedentary behavior. Given the strong relationships between sedentary behavior, PA and health in older adults, programs are needed to address these behaviors. A better understanding of sex and age differences in sedentary behavior and PA in older adults can inform interventions and guide public health messages.

## Data Availability

The datasets used during the current study are available from maria.wahlberg@ki.se, on reasonable request.
